# Post-circumcision penile skin loss: reporting the outcome of one-stage anterolateral scrotal based flaps in children

**DOI:** 10.1080/2090598X.2022.2146835

**Published:** 2022-11-16

**Authors:** Abdelqawey Yousef, Salah Nagla, Mohamed Fathy, Mohamed Negm

**Affiliations:** a Department of Genital Reconstructive Surgery, Sheikh Zayed Private Hospital, Cairo, Egypt; b Urology Department, Tanta University, Tanta, Egypt; c Pediatric Surgery Unit, Minia University, Minia, Egypt; d Pediatric Surgery Unit, Qena Faculty of Medicine, South Valley University, Qena, Egypt

**Keywords:** Penile skin loss, circumcision, scrotal flap

## Abstract

**Introduction:**

Improper penile assessment, together with carrying out circumcision by an inexperienced person, results in major complications. One of the complex complications is the complete or sub-complete penile skin loss, which in many cases, necessitates one or staged repair.

**Purpose:**

To evaluate modified one-stage bilateral anterolateral scrotal-based flaps to compensate for penile skin loss after circumcision.

**Methods:**

This study was performed on patients with almost penile skin loss after circumcision from February 2013 to July 2021. In all cases, one-stage modified bilateral anterolateral scrotal skin flaps were used to compensate for penile skin loss. The modification includes scrotal skin flap fashioning in a novel way, in addition to the use of penodermal fixation sutures at the penoscrotal junction, to create a stable penoscrotal junction and new penile skin coverage. Patients were discharged from the hospital on the same day of surgery. The dressing was left for 5 days. Follow-up visits were scheduled weekly in the first month, 3 and 6 months later, then annually.

**Results:**

Forty-six children were included in this study. Their mean age was 4.5 ± 1.5 years. The mean operative time was 139.6 ± 11.5 min. No flap ischemia or necrosis was reported. One case (2.2%) developed a scrotal hematoma managed conservatively. Three (6.5%) cases presented with wound dehiscence at the penoscrotal angle. Three (6.5%) cases had self-limited penile edema. Two (4.3%) cases had dorsal midline hypertrophic scar; one improved after treatment with triamcinolone acetonide ointment, and the other needed scar revision. The mean follows up was 23.33 ± 9.13 months.

**Conclusion:**

The modified scrotal skin flap technique provides a good substitution for stable penile skin coverage and a one-stage reconstruction of penile skin loss. It results in good parents’ satisfaction with acceptable complications.

## Introduction

The penile skin is a unique form of skin because it accommodates penile erections and sexual frictions. Moreover, the normality of external genitalia is important for male self-esteem [1]. Circumcision is still practiced by non-experienced persons, especially in underdeveloped countries [2,3]. Many complications may occur after circumcision, including different degrees of penile skin loss. These complications occur due to improper surgical planning and undiagnosed penile anomalies, especially buried and webbed penis [4,5]. Penile skin loss after circumcision mandates a substitution, whether with a graft or flaps. Many authors have reported their results with split and full-thickness skin grafts, while others have used one- or two-stage skin flaps [6–8]. In the literature, few studies have reported using scrotal skin flaps for compensation for penile skin loss after circumcision in a pediatric population. This study presents our experience in modified one-stage bilateral anterolateral scrotal-based skin flaps to compensate for penile skin loss after circumcision in children.

### Patient and methods

A patient database maintained by the authors for boys with almost penile skin loss after circumcision was retrospectively collected and analyzed. This is a case series (Level IV Evidence). The data was collected during the period from February 2013 to July 2021. Ethical committee approval was granted for data collection. The study follows the principles of the Declaration of Helsinki and its later modifications. Before surgery, informed consent was assigned from the parents after explaining all related hazards of the operation.

### Study cohort

**Figure 1.**
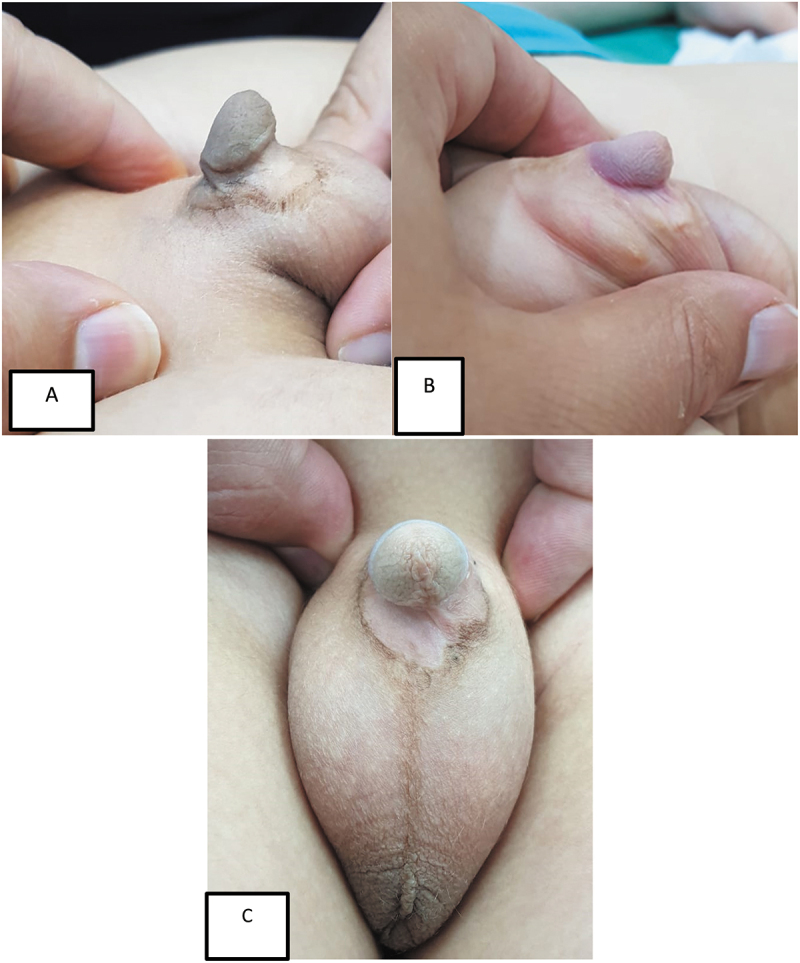
Different presentations of post-circumcision penile skin loss.

Patients with almost penile skin loss after circumcision were included (Figure 1). Cases with penile skin loss due to other causes rather than circumcision or associated with the urethra or glans injuries were excluded. The following were done for all cases: Full history taking and thorough clinical examination; history of previous procedures; outcome assessment with both surgeon and parental satisfaction; and complications and the need for another stage

### *Surgical technique* (Figure. 2)

**Figure 2.**
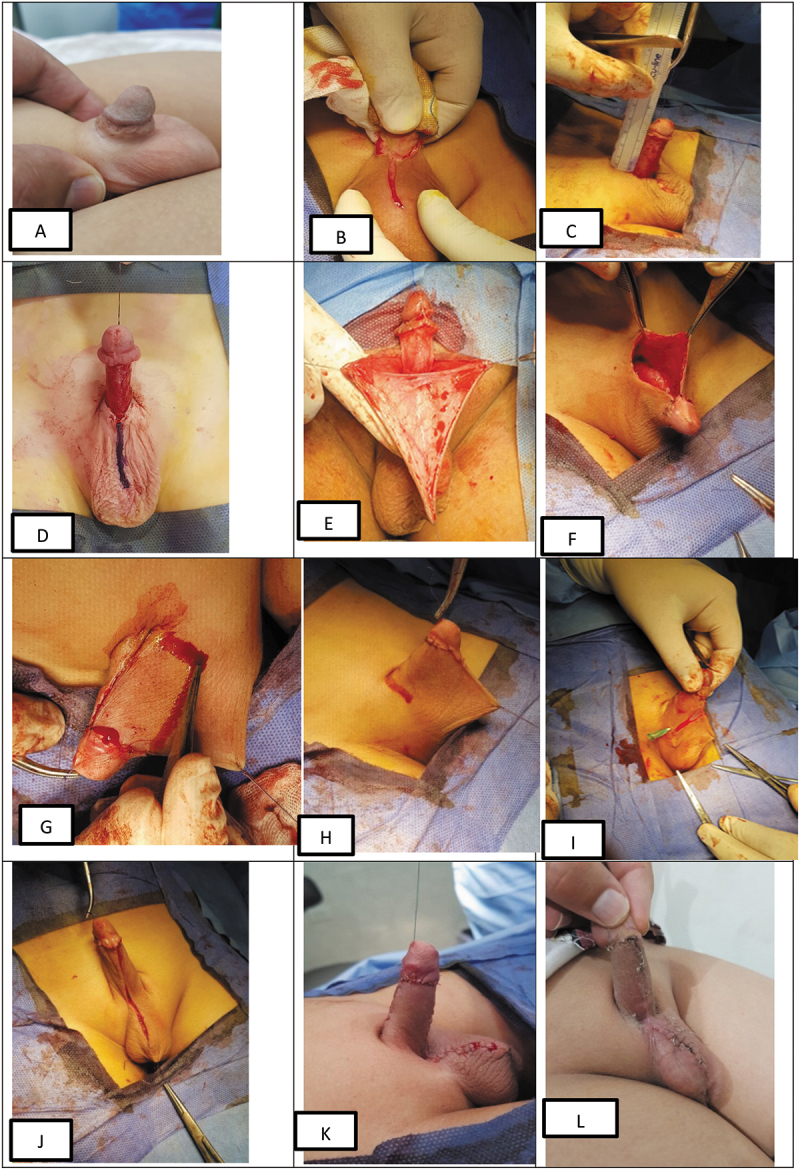
Detailed operative steps of the technique.

Under general anesthesia with caudal analgesia, a traction suture was taken at the glans. A circumcising incision proximal to the coronal sulcus was then performed (Figure 2(b)). The dissection plane was between Buck’s fascia and the penile skin with excision of fibrosis and abnormal dartos fascia. We rarely inserted a urethral catheter during penile degloving in cases of dense periurethral adhesions. The length and girth of the degloved penis were then measured to fashion a suitable scrotal flap (Figure 2(c)). A midline scrotal incision started the preparation of the flaps at the median raphe from the penoscrotal junction downwards (Figure 2(d)). The length of this incision was equal to the length of the degloved part of the penis (Figure 2(e)). Next, the penis was buried in the scrotum while stretched to ensure the needed length of the flap (Figure 2(f)). The median scrotal incision was then closed at the dorsum of the penis (Figure 2(g)). The scrotal skin was wrapped around the penis, mimicking the first stage Cecil-Culp scrotal flap. Dorsal midline scrotal skin closure was accomplished from the penopubic junction to the corona using 5/0 or 6/0 Vicryl. Upward traction of the penis was then performed to fashion the flaps by inverted V-shaped marking (Figure 2(h, i)). At this step, we should maintain a wide flap base around the penis without tension. The two lateral incisions (two limbs of inverted V) were performed with downward migration of the remained parts of the scrotal skin mimicking scrotal skin harvesting in the second stage of Cecil-Culps’s scrotal flap (Figure 2(j)). The stable penoscrotal angle was recreated by fixing the new penile skin using PDS or polyproline 5/0 sutures as penoscrotal Fixation sutures at 4–8 o’clock lateral to the urethra. These fixation sutures prevent the development of buried and webbed penis after scrotal skin flaps. Later, the ventral parts of both flaps were approximated at the midline and sutured to each other to cover the ventral part of the penis (Figure 2(j, k)). Closure of the scrotal wound was then accomplished. The urinary catheter, if any, was removed after completion of the procedure and before the patient was ambulated from the operation room. A non-tight dressing over a local antibiotic ointment was applied around the penis, and the dressing was left for 5 days.

### Postoperative care

The patients were discharged on the same day of surgery. Dressing removal was done on the fifth postoperative day, then parents were instructed for local wound care, with continuous communications between follow-up visits. Follow-up visits were scheduled weekly for one month (Figure 2(l)), then 3 and 6 months later, then annually.

### Outcome assessment

The primary outcome was the Presence of healthy skin covering the whole penile length from the penile base to the coronal sulcus. Healthy skin does not have hypertrophic scars or contractures. Complications were the secondary outcome, including; skin wound dehiscence, wound infection, penile edema, hematoma, and penile curvature with/without the penile rotation. Follow-up visits were then scheduled weekly for one month, then 3 and 6 months later, then annually. At the sixth-month visit, the parents’ satisfaction was determined by an independent surgeon based on the following scale: (grade 0) ‘Very unsatisfied’, (grade 1) ‘Unsatisfied’, (grade 2) ‘Neither satisfied nor unsatisfied’, (grade 3) ‘Satisfied’, (grade 4) ‘Very satisfied’ [9].

## Results

**Figure 3.**
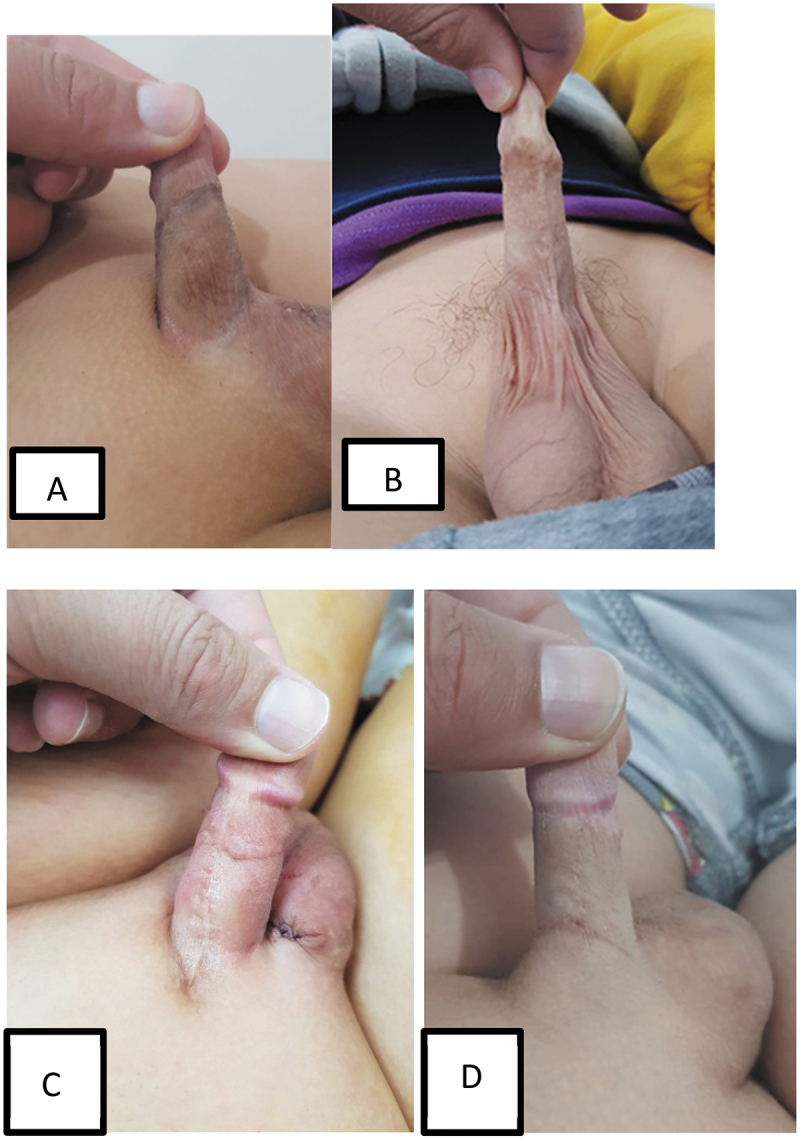
Outcome at the postoperative follow-up after 6 months. (a) Preoperative. (b, c) Immediate postoperative photos of dorsal and ventral suture line. (d, e) Follow up after two years with good looking scar.

This study included 46 patients; their mean age was 4.5 ± 1.5 years. All cases have reported previous circumcision (Table 1). The mean operative time was 139.67 ± 11.52 min (120–160 min). The scrotal drain was left in ten (21.7%) patients. Penile skin coverage with preservation of penoscrotal angle was achieved in all cases (100%). Good results were obtained during patients’ follow-up (Figure 3), but minor self-limited complications occurred in nine (19.56%) cases (Table 2). Partial wound disruption at the neo-penoscrotal angle developed in three (6.5%) cases managed conservatively. Penile edema for more than 2 weeks occurred in three (6.5%) patients and resolved with time in all patients. Two patients presented with hypertrophic scar (one improved after local application of triamcinolone acetonide, and the other needed scar revision). Self-limited scrotal hematoma occurred in one (2.2%) case. We neither reported flap necrosis nor contracture. The mean follows up was 23.33 ± 9.13 (6–40) months. The patient’s satisfaction was assessed using a 0–4 score (Table 3) obtained from the parents at the 6-month visit. The mean patients’ satisfaction score was 3.26 ± 0.68 (1–4). The primary outcome was achieved in 45 (97.8%) out of the 46 cases.

**Table 1.** Patients’ presentation.

Previous circumcision	No. of Patients	Percentage (%)
**OnceTwiceThrice**	28	60.9%
	12	26.1%
	6	13%

**Table 2.** Post-operative complications.

	No. of Patients	Percentage
**Scrotal hematoma**	1	2.2%
**Penile edema**	3	6.5%
**Skin dehiscence**	3	6.5%
**Hypertrophic scar**	2	4.3%

**Table 3.** Parents’ satisfaction score.

Grade		Frequency	Percent	Mean(SD)
Valid	**0**	0	0	
	**1**	1	2.2	
	**2**	3	6.5	3.26 ± 0.68
	**3**	25	54.3	
	**4**	17	37.0	
	Total	46	100.0	

## Discussion

Penile skin loss can happen with mono-polar diathermy during circumcision, after penile skin tumors, or following severe burns [8,10]. This situation occurs more frequently during circumcision, especially if a non-qualified person performs it. The skin deficiency is augmented with missed congenital penile anomalies at the time of circumcision (e.g. buried penis, webbed penis). Ventral skin deficiency after circumcision usually results from circumcision neglecting the anomaly of the penoscrotal web; this can be corrected by a ventral skin lengthening procedure, as described by Negm and Nagla [5]. The magnitude of the problem increases with faulty repeated circumcision in the same patient. Various degrees of penile skin deficiency could be presented after circumcision. However, there is no consensus in the current literature about repairing partial penile skin loss. In this study, patients with penile skin loss were included. Cosmetic appearance and psychological concerns were children’s and parents’ main concerns. Abbas et al. reported that the main complaints of patients with trapped penis were cosmetic (60%), voiding (56.6%), and psychosocial concerns (50.5%) [11]. Penile shaft skin compensation is accomplished either by skin flaps or grafts. Non-hirsute skin grafts are among the most popular methods, and their uptake depends on the local tissue condition of the recipient area, which is questionable in many situations. At the same time, skin grafts are less elastic and offer less resistance during sexual intercourse. Moreover, grafts are more vulnerable to retraction with restriction of the filling of the corpus spongiosum [4]. Full-thickness skin grafts have better skin compensation but a higher liability for graft rejection [12,13]. Many skin flaps are available to compensate for penile skin, like axial pattern skin flaps. However, these flaps give the penis a bulky appearance [14].

This study’s choice of scrotal flaps was based on the many advantages of the scrotal flaps as they lack subcutaneous fat, have high elasticity, and have a rich blood supply [15]. The scrotal skin also has a close anatomic relation to the penis. In 1950, Goodwin and Thelen [16] studied the multistage compensation of the penile skin utilizing the scrotal skin. The resultant multiple scars limited this surgery. Fakin et al. [7] used a bipedicled anterior scrotal flap, which could compensate for most penile skin loss after silicon injection in adults. These authors concluded that penile skin defects could be covered by a well-vascularized scrotal flap with good functional and cosmetic outcomes. Although scrotal skin has an abundant blood supply and good sensory innervation, it is a hirsute skin [17–19]. This problem of hairy scrotal flaps could be solved by hair epilation if it was annoying to the patient [20]. Currently, scarce data has been published addressing the use of scrotal flaps in children after circumcision.

The mean age of patients was 4.5 ± 1.5 years, whereas the mean age at the first circumcision was 2.02 ± 0.52 months. The long-time lag between circumcision and reconstruction (mean 9.33 ± 2.6 (2–73 months) is usually secondary to the wrong belief in a large percentage of parents that the problem is in the penile size, which will be corrected with age. This study’s mean operative time was 139.67 ± 11.5 (120–160) min. Fakin et al. encountered minor complications in 40% of their patients: they had partial necrosis in 9%, hematoma in 12%, and wound dehiscence in 19%. In this study, minor complications occurred in nine cases; scrotal hematoma in one (2.2%), partial wound disruption at the neo-penoscrotal angle in three (6.5%) cases, and self-limited penile edema in three (6.5%) patients. This could be explained by the meticulous dissection, the use of bipolar diathermy to control bleeding, and a non-tight dressing [7]. The difference in the complication rates between this study and Fakin’s study could also be explained by different patient populations (adults). It could be assumed that the complication rate could be higher due to larger defects and the need for extensive tissue mobilization to cover the resulting defect. Also, adults have higher vascularity, which can increase the risk of hematoma and penile edema. Further, erections in adult patients can increase the risk of complications, including wound dehiscence. Guo et al. [8] used scrotal flaps to cover the deficient penile skin after burn injuries in 17 adult males with good results. They had three cases of wound dehiscence, which were later covered by skin grafts. This high incidence of wound dehiscence might be attributed to the local skin condition after the burn and might not relate to the technique itself. Also, skin dehiscence, especially at the penoscrotal angle, could be secondary to tension sutures that might occur with the bi-pedicled flap. In the present study, using one-stage scrotal flaps decreases the number of procedures needed to achieve a suitable length and aesthetic penile appearance. The mean follows up was 23.33 ± 9.13 (6–40) months. There was no reported ventral scar because the bilateral flaps were ventrally closed at the midline, mimicking the median raphe of the penis. Keloid scar was reported twice at the dorsal suture line; one was improved by anti-scar ointment, while the other needed operative scar removal. Moreover, we overcame the problem of skin disruption by fashioning a wide-based flap. In addition, we used to preserve a new penoscrotal angel by hidden penodermal fixation sutures between the penile tunica and the scrotal dermis. In addition, Kim et al. [21] used the bi-pedicle scrotal flap in five adult males after penile skin loss due to paraffin injection. These authors reported good patient satisfaction after the procedure with good penile skin coverage with no penile skin shortage. Guo et al. [8] used a 1–5 score, which measured cosmetic satisfaction as follows; 1 denoted Very dissatisfied, 2 denoted Moderately dissatisfied, 3 denoted Equally satisfied and dissatisfied or accepting the results, 4 denoted Moderately satisfied, and 5 denoted Very satisfied. They stated that 85% of their patients were satisfied with the morphology. At the same time, Fakin et al. [7] had a median patient satisfaction score of 4.37. In the present study, the mean patients’ satisfaction score was 3.26 ± 0.68 (1–4) on a score from (0–4). This parents’ satisfaction score was 1 in one patient, 2 in three, 3 in 25, and 4 in 17. In our study, most of the parents were satisfied with the technique. The known technique of the lateral scrotal flaps depends on separating two lateral scrotal skin flaps after measuring the penile girth and length. Next, the two flaps are approximated at the ventral and dorsal aspects of the penis. Our modified technique aims to fashion the scrotal skin flaps around the penis before separating the flaps from the scrotum. This provides an exact estimation of the flap around the penis, and it avoids under or overestimation of the flap due to additional girth of the dartos layer under the flap. In addition, this technique avoids the tension sutures that might happen with the bipedicled flap at the penoscrotal angle. Moreover, the technique allows adding fixation sutures to create a new stable penoscrotal angle – as described by Negm et al. [22] to avoid the common drawbacks of the scrotal flaps, e.g. buried and webbed penis. Moreover, compensation for penile skin loss in a one-stage procedure decreased the surgical trauma and emotional and financial burden on the family and medical resources.

### Limitations to this study

Although this was a retrospective study, digital data archiving has ameliorated its drawbacks. There is no comparative arm in this study, which is a skin graft; however, in our experience, skin grafts have more functional problems than scrotal flaps, which directed our practice for reconstruction using this modified technique. Scrotal skin is hair-bearing, and there is a visible dorsal suture line/potential scar on the dorsal aspect of the penis outside the circumcising incision. Although visible dorsal scars were infrequently encountered among our patients, it seems to be another limitation of this technique, so long-term assessment is still required.

## Conclusion

The one-stage bilateral modified anterolateral scrotal skin flaps technique is suitable to compensate for penile skin loss after circumcision at pediatric age with minor self-limited complications. In addition to proper penile skin coverage, the technique could maintain a new penoscrotal angle with minimal ventral and dorsal scars.
